# Evaluating a novel patient pathway to manage symptomatic breast referrals (the blue flag clinic): a longitudinal observational study

**DOI:** 10.1308/rcsann.2023.0028

**Published:** 2023-07-25

**Authors:** TJE Hubbard, X Liu, M Sulieman, P Drew, I Brown, R English, I Abbas, K Potiszil, M Barta, N Jackson, P King

**Affiliations:** ^1^ Royal Cornwall Hospitals NHS Trust, UK; ^2^ University of Exeter, UK

**Keywords:** Neoplasia, Breast, Oncology

## Abstract

**Introduction:**

A novel referral pathway for exhibited breast symptom (EBS) referrals to manage increasing referrals of urgent suspected cancer (USC) was implemented in our trust. We report on the safety and effect on compliance with the 2-week-wait rule (2WW).

**Methods:**

A single-centre longitudinal observational study included all patients referred to a UK breast unit during 13 May 2019 to 27 March 2020 (period 1) and 8 February 2021 to 31 January 2022 (period 2). USC referrals were assessed in a one-stop clinic (red flag clinic [RFC]); EBS referrals were assessed in a new clinic in which clinical evaluation was performed and imaging occurred subsequently (blue flag clinic [BFC]). Patients were followed up to determine the symptomatic interval cancer rate.

**Results:**

There were 9,695 referrals; 1,655 referrals (17%) were assessed in the BFC after 63 exclusions. Some 95.9% of patients had a benign clinical examination (P1/P2), 80.1% had imaging (mammogram or ultrasound) and 4% had a tissue biopsy. In total, 16/1,655 (0.97%) BFC patients and 510/7,977 (8.2%) RFC patients were diagnosed with breast cancer (breast cancer detection rate). Some 1,631 patients (with 1,639 referrals) were discharged and followed up for a median of 17 months (interquartile range 12–32) with one subsequent cancer diagnosis (symptomatic interval cancer rate, 0.06%). Implementation of the BFC pathway increased 3-month average trust performance of USC referrals with 2WW standard from 8.5% to 98.7% (period 1) and from 30% to 66% (period 2).

**Conclusions:**

The BFC pathway for EBS patients is safe and implementation led to improvement against the 2WW target for USC referrals, ensuring resources are prioritised to patients with the highest likelihood of breast cancer.

## Introduction

Breast cancer is the most common cancer in the UK;^1^ UK cancer survival lags behind that in other European countries and early diagnosis is a priority for the National Health Service (NHS).^2^ Despite the NHS Breast Screening Programme, the majority of breast cancers are still diagnosed via the symptomatic route.^3^ These referrals are ‘urgent suspected cancer’ (USC) if the symptom, age and gender of the patient fulfils National Institute for Health and Care Excellence (NICE) referral guidelines because they are deemed ‘high risk’ with a positive predictive value (PPV) of cancer of over 3%.^4^ If symptoms and demographics do not match the NICE criteria it is an ‘exhibited breast symptom’ (cancer not initially suspected) (EBS) referral. The current NHS England performance target is that 93% of these patients (both USC and EBS referrals) should be seen within a ‘2-week wait’ (2WW).^5^ In practice, the majority of breast units assess patients from both the USC and EBS referral pathway in a one-stop clinic where clinical assessment and radiological imaging (mammogram and/or ultrasound) occur on the same day.

There has been a year-on-year increase in the numbers of patients referred to the breast clinic, with a concomitant decrease in the ‘conversion rate’ (proportion of referrals that result in a cancer diagnosis).^3^ In July 2022, only 74% of patients referred were seen within 2 weeks in England,^6^ leading to potential delayed diagnosis and treatment in this USC cohort.

There is a national shortage of breast radiologists and imaging availability is often a bottleneck for one-stop clinic capacity.^7^ Identifying patients at low risk of cancer and uncoupling imaging from the clinical evaluation – or not imaging those at very low risk – could improve the time to assessment for those at higher risk. A number of new pathways based on this premise are being implemented across the UK, mostly focusing on breast pain.^8–10^ However, these pathways are relatively new, assess only breast pain and are currently undergoing evaluation.

In our trust, a new pathway was implemented in 2019 to allow referring primary healthcare professionals to self-triage referrals. Patients referred via USC criteria were seen in a one-stop clinic (red flag clinic [RFC]), whereas those referred via EBS criteria were seen in a novel clinic where clinical evaluation and imaging (if required) is uncoupled (blue flag clinic [BFC]). This is a retrospective observational study reporting this novel pathway, demonstrating the outcomes of those referred to the BFC and assessing its impact on trust performance in meeting the 2WW target for USC referrals.

## Methods

### Data collection

This study was a retrospective analysis of prospectively gathered data of referrals from primary care to a single UK breast unit, approved by the local audit and service provision department (registered audit number 2413).

Hospital databases were manually interrogated for patient information on symptoms, imaging and outcome (e.g. cancer). Patients were assessed by clinical evaluation and imaging, and a grade noted for how suspicious for malignancy the assessment is. This is graded 1 (normal, low suspicion of malignancy) to 5 (definite malignancy) and used for clinical evaluation (P), mammogram (M) and ultrasound (U).

Follow-up was by review of patient hospital records up to 30 July 2022, including matching the patient unique identifier against the local breast cancer registry database to ascertain whether the patient had a subsequent breast cancer diagnosis.

### Description of pathways

**Figure 1:**
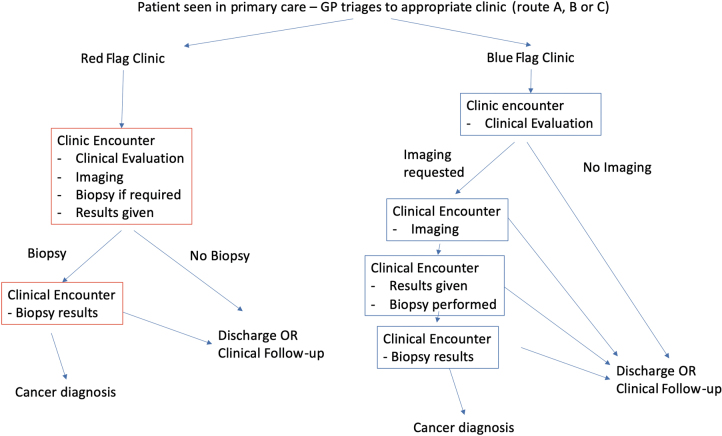
Schema of patient flow through the red flag and blue flag pathway

Patients were seen by a primary care healthcare practitioner, referred using either the USC or EBS criteria and automatically triaged to either the RFC or BFC. All patients on the USC pathway were seen in the RFC. EBS referrals were seen preferably in the BFC; however, because there was insufficient capacity in the BFC to accommodate all EBS referrals, patients who could not be booked into the BFC were seen in the RFC. The referral pathways are shown in Figure 1 (referral criteria are given in Supplementary Figure 1 available online). Both the RFC and BFC are staffed by breast consultants, middle grade doctors and general practitioners (GPs) with a specialist interest in breast surgery who are competent in the assessment, investigation and treatment of breast disorders. The target is that all patients are seen within 2 weeks from referral. The clinics differ in their imaging pathways; in the RFC, imaging appointments and slots are linked to the clinic, requiring surgeon and radiology staffing and availability to match to the same time.

In the BFC, imaging is uncoupled from the clinical evaluation. The patient returns on a separate day (within 2 weeks) for an imaging appointment. This allows surgeon and radiology availability to be uncoupled, and a ‘session’ of radiology to be fully utilised and booked in advance. The ‘clock’ stops after the initial consultation, because this is the date when the patient is seen for the first time following the referral receipt (a metric measured by NHS England).^5^

### Inclusion criteria

All symptomatic referrals to the Royal Cornwall Hospitals NHS Trust Mermaid Breast unit for clinical evaluation during the periods 13 May 2019 to 27 March 2020 and 8 February 2021 to 31 January 2022 on either the USC or EBS referral pathway were included. There was a break in the running of the referral pathways between 28 March 2020 and 7 February 2021 due to the COVID-19 pandemic when different pathways to limit patient contact were implemented. Referral episodes were counted rather than individual patients, because each referral episode was a discrete assessment.

### Exclusion criteria

Referrals with an appointment not attended by the patient were excluded. Referrals seen in the BFC, but not for a new presentation (i.e. had been booked into BFC appointment for administrative purposes, but were already under the care of the breast unit) were excluded. There were no other exclusions.

### Primary outcome

The primary outcome was to assess the safety of the BFC pathway. The ‘symptomatic interval cancer rate’ (analogous to the NHS Breast Screening Programme interval cancers^11^) was measured by reporting how many patients had been initially seen in the BFC and discharged as not cancer, who were subsequently diagnosed as having breast cancer. These patients were reviewed individually, and it was determined whether it was a true ‘missed cancer’. This metric has been used previously to determine the safety of symptomatic breast cancer assessment pathways.^12,13^ Patients were followed up from discharge to 1 July 2022.

### Secondary outcome

Information on the presenting symptoms, investigations, time to investigation, follow-up appointments and outcome was obtained and reported for the novel BFC pathway. Number of referrals and outcome of cancer vs discharged not cancer for referrals seen in the RFC are reported as a comparator. Disease diagnosis was defined by International Classification of Diseases (ICD) codes: C50.0–C50.9 (i.e. malignant neoplasm of the breast). Any other ICD classifications were classified as ‘other cancers’. The outcomes of referrals for breast pain only underwent subgroup analysis, owing to increased national interest in creating ‘breast pain only’ clinics.

As a measure of clinical effectiveness, and ability to reduce waits for USC patients, the impact of the introduction of the BFC pathway was assessed by retrospective analysis of the trust’s performance in achieving the percentage of patients seen within 2 weeks of referral on both the USC and EBS pathways. Data were obtained from NHS England monthly provider cancer waiting time statistics to give the monthly performance of the trust in achieving the national target for USC and EBS referrals to be seen within 2 weeks of referral.^14^ Average percentage of patients seen within 2 weeks for the 3 months before the pathway implementation was compared with the same figure for the 3 months after implementation.

Missing data were checked manually with the digital systems. Information that remained missing (e.g. clinical evaluation P value) is recorded as ‘not specified’.

This study report conforms to the STROBE guidelines (Supplemental information available online).^15^

### Statistical analysis

Information was collected using Excel (Microsoft, Redmond, WA, USA) and statistical analysis performed on SPSS v26 (IBM, Armonk, NY, USA).

Demographic data are described as proportions and percentages. Continuous data are described as mean ± standard deviation (sd) and discrete data as median ± interquartile range (iqr). The PPV of the presenting symptom for predicting a diagnosis of breast cancer was calculated as Number of true positives (presenting symptom with cancer)/Number of true positives + Number of false positives (all referrals with presenting symptom).

## Results

### Cancer detection rate

**Figure 2:**
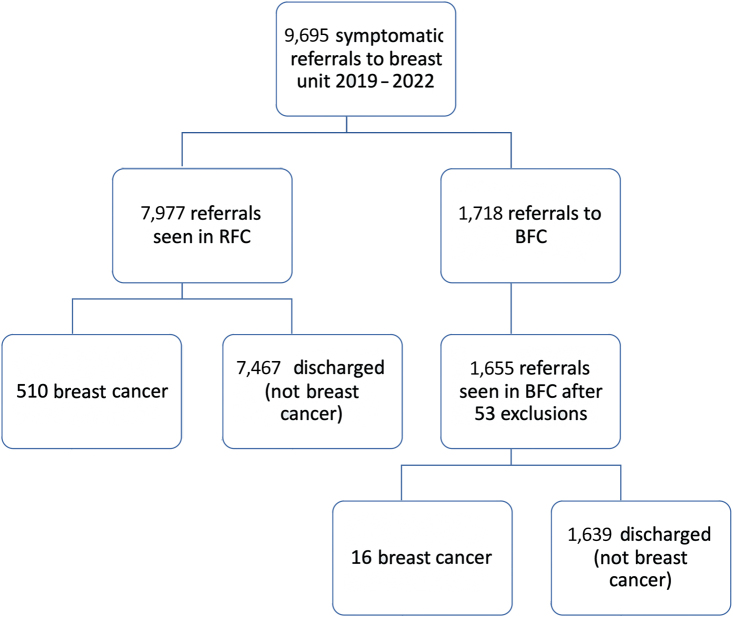
Flow diagram of all referrals to the breast unit for clinical evaluation during the study period, showing the diagnostic outcome of included patients.BFC = blue flag clinic; RFC = red flag clinic

There were 9,695 referrals to the breast unit for clinical evaluation during the study period. Figure 2 presents a flow diagram demonstrating the outcome following referral.

Some 7,977 referrals were seen in the RFC and 510 breast cancers diagnosed, giving a cancer detection rate of 6.39%. Of the 7,977 referrals, 5,914 (74%) were USC referrals with 486 having an outcome of breast cancer (8.2% cancer detection rate), and 2,063 (26%) were EBS referrals seen in the RFC because of insufficient capacity in the BFC, with 24 having a breast cancer outcome (1.16% cancer detection rate).

Some 1,718 referrals were seen in the BFC; 53 referrals (36 patients) were excluded because they did not attend their appointment and 10 referrals were excluded because they were currently undergoing treatment by the breast service. After exclusions, 1,655 referrals (1,631 patients) were included in the detailed analysis; 24 were re-referrals (re-referral rate of 1.45%). Of note, during the study period, no patients who were seen in the BFC were concurrently or subsequently seen in the RFC. Of the 1,655 referrals seen in the BFC, 16 breast cancers were diagnosed, giving a BFC detection rate of 0.97%.

### Outcomes of the BFC pathway

The mean age of patients seen in the BFC was 45 years (sd 18) and 1,386/1,655 (83.7%) were female. The assessment and evaluation outcome of patients is given in Table 1. The majority of referrals had benign clinical findings (P1 or P2 accounted for 95.9% of patients). In total, 80.1% of referrals had some form of imaging, either mammogram or ultrasound. Median time to imaging was 1 day (iqr 0–7) for mammograms and 9 days (2–16) for ultrasound. Some 141 referrals (8.5%) had a follow-up clinic appointment (either face-to-face or telephone); median time to follow-up was 38 days (iqr 24–64). Sixty-six referrals (4%) had a biopsy, 16 (24%) of which were diagnosed with breast cancer and 7 (10%) were diagnosed with a non-breast cancer. These presentation and investigation characteristics did not vary between the two time periods of data collection (Supplementary Table 1 available online).

**Table 1:** The triple assessment (clinical, radiological and pathological) findings of the referrals to the blue flag clinic

Clinical finding	Number	Percentage
P1	843	50.9
P2	744	45
P3	26	1.6
P4	4	0.2
P5	1	0.1
Not specified	37	2.2
Had imaging	1,325	80.1
Had mammogram	803	48.5
M1	514	31.1
M2	253	15.3
M3	27	1.6
M4	4	0.2
M5	4	0.2
No mammogram	852	51.5
Had ultrasound	759	45.9
U1	418	25.3
U2	301	18.2
U3	22	1.3
U4	9	0.5
U5	5	0.3
No ultrasound	896	54.1
Had biopsy	66	4.0
Breast cancer diagnosed	16	24
Other cancer diagnosed	7	10
Benign biopsy	43	65

The presenting symptoms of referrals to the BFC were analysed, and the PPV of the presenting symptom being diagnosed with a breast cancer was calculated (Table 2). Nipple pathology had the highest PPV (1.96%) for cancer detection, followed by lump (1.3%) and pain (0.76%), with other presenting symptoms (skin changes, lumpiness and other symptoms) not resulting in a cancer diagnosis.

**Table 2:** Positive predictive value of presenting symptom to the blue flag clinic and cancer detection

Symptom	Number referred	Percentage	Number with cancer	PPV
Lump	689	41.6	9	1.3
Pain	654	39.5	5	0.76
Skin changes	44	2.7	0	0
Lumpiness	68	4.1	0	0
Nipple pathology	102	6.2	2	1.96
Other	98	5.9	0	0

PPV = positive predictive value.

### Breast pain sub-analysis

The 654 referrals presenting with breast pain as the primary symptom were cross-referenced with the clinical evaluation P values (Table 3). In total, 649 (of 654) had a benign clinical evaluation of P1 or P2, of these 2 (of 649) had a subsequent diagnosis of breast cancer on imaging. Therefore, the PPV in patients presenting with breast pain with a benign clinical evaluation was 0.3%.

**Table 3:** Clinical evaluation findings (P value) and breast cancer detection outcome in referrals to blue flag clinic with breast pain as presenting symptom

P value	Not breast cancer	Breast cancer	Total
Not specified	11	0	11
P1	531	2	533
P2	105	0	105
P3	2	3	5

### Symptomatic interval cancer rate

In total, 1,615 patients (with 1,639 referrals) were discharged from the BFC with a non-cancer diagnosis. The median follow-up from discharge was 17 months (iqr 12–32). One of 1,639 referrals was subsequently diagnosed with breast cancer, giving an interval symptomatic cancer rate of 0.06%.

The case was reviewed; the patient was a male diagnosed clinically with gynaecomastia (P2) and had imaging of a mammogram showing gynaecomastia only (M2). One year later he re-presented to clinic with a separate mass associated with gynaecomastia and imaging and biopsy subsequently confirmed a breast cancer.

### Impact on 2WW targets

The impact of introduction of the BFC pathway on 2WW target achievement is displayed in Supplementary Figure 2 (available online). This clearly shows that implementation of the BFC pathway improved the trust’s performance against the 2WW target for USC referrals at first implementation, going from an average of 8.5% of referrals being seen in 2 weeks in the 3 months prior to implementation to 98.7% in the 3 months after implementation. This was a sustained improvement throughout the whole of the first implementation period, during which the percentage of EBS referrals seen within the 2WW target was high (>90%).

At the second implementation, during the 3 months prior to implementation an average of 30% of USC referrals were seen within 2 weeks; an average of 66% were seen within 2 weeks during the 3 months after implementation. This included April 2021, during which only 12% of USC referrals were seen within 2 weeks; this improved to 98% by June 2021, before declining again between October 2021 and January 2022. During the second implementation, the percentage of EBS referrals seen within the 2WW target was consistently low and remained <70% throughout.

## Discussion

We present a novel BFC pathway to investigate breast symptoms referred on the EBS pathway. There are precise referral criteria, which are auto-triaged by the primary care practitioner obviating the need for laborious secondary care triage. The BFC pathway assessed a large proportion of patients referred to the breast unit and the percentage of USC patients seen within 2 weeks increased. Crucially, the BFC pathway is safe, with only one ‘interval cancer’.

An important finding is the relatively low rate of imaging in the BFC. Some 48.5% of referrals had a mammogram and 45.9% had an ultrasound, with 19.9% of patients having no imaging at all. This allows increased efficiency of radiology time to ensure an imaging clinic can be fully utilised, rather than having a radiologist available – but not necessarily utilised – within a ‘one-stop’ clinic.

The BFC was a highly utilised pathway receiving 17% of symptomatic referrals. However, 39% of symptomatic referrals were EBS criteria and could have been seen in the BFC. Now that the BFC has been shown to be safe, its capacity has doubled and we expect all EBS referrals to be seen in the BFC.

The BFC pathway was established to manage patients according to the NHS England 2WW target. During the first implementation period, USC referrals meeting the 2WW target went from 8.5% pre-implementation to 97.5% post-implementation. In the second implementation period, USC referrals meeting the 2WW target went from an average of 30% pre-implementation to 96% in the first month post-implementation, although this was not sustained with a 3-month post-implementation average of 60%. This was due local factors of staff shortages and continued service disruption post COVID-19.

The Faster Diagnosis Standard (FDS) will shortly replace the 2WW standard as a metric of cancer diagnosis. The FDS measures the percentage of USC patients given a diagnosis within 28 days of referral.^16^ This still requires streamlined and effective assessment pathways and the BFC will remain relevant.

Symptom PPVs within the BFC were all below the current NICE threshold for USC of 3%. Some symptoms (notably ‘lumpiness’) had no patients who were subsequently diagnosed with cancer. The finding that breast pain with a normal examination has a low incidence of breast cancer of 0.3% is concordant with other studies.^17^ A benefit of the BFC pathway compared with ‘breast pain only’ referral pathways is that a wider range of symptoms (and therefore number of patients) can be managed via the pathway. The overall cancer detection rate within the BFC was below 1%. This confirms that patients referred on the EBS criteria have a very low risk of being diagnosed with breast cancer.

The symptomatic interval cancer rate in the BFC was 0.06%. The one subsequent cancer presented as a lump on the ipsilateral side to the initial presenting symptom, and is likely be classified as a ‘missed cancer’. This interval symptomatic cancer rate is lower than the previously reported missed cancer rate of 0.17%^13^ and the same as a subsequent report of 0.06%.^12^ It is also below the interval cancer rate in the NHS Breast Screening Programme of 0.3%.^18^

### Limitations of the BFC pathway

The percentage of EBS referrals seen within 2 weeks was consistently below the USC referrals, and subsequent time to imaging was a median of 10 days. However, the BFC breast cancer detection rate (0.97%) is only marginally above the screening cancer detection rate (0.8%),^19^ in which patients wait 3 years between screening mammograms. In this BFC group with a very low cancer detection it may be appropriate to relax stringent targets (such as 2WW or FDS) to prioritise those with USC symptoms (cancer detection rate 8.2%) in a healthcare system of finite resources.

A limitation of the BFC pathway is the inconvenience of returning at a later date for patients requiring imaging. Mammograms were generally performed on the day of clinical review, but reported subsequently; therefore, only the 45% of those who had an ultrasound needed to return. Other potential difficulties could be a lack of radiology slots and ensuring that all imaging is performed within 2 weeks. In our experience, the ability to plan the radiology clinics in advance with knowledge of how many patients require scanning meant that clinics were very time efficient and lack of capacity was not an issue, which is in stark contrast to the one-stop model in which radiology scheduling is difficult to anticipate and can be quickly overwhelmed.

Once the pathway is established, referrers may bypass the slower assessment route of an EBS referral and send all referrals as USC, which has occurred in other breast pathways.^20^ However, the re-referral rate to the BFC of 1.45% is lower than the reported re-referral rate to the one-stop clinic (15%–31%),^12,13^ and no patients seen in the BFC were subsequently referred via the USC criteria within the study period. This suggests patient and referrer satisfaction with the process; however, we are considering formal patient satisfaction surveys as part of our ongoing evaluation of the pathway.

### Limitations of study

There are some limitations of this service evaluation study. Data were collected retrospectively, with some missing values (e.g. for P values); however, these do not adversely affect our main findings. Similar clinical details for RFC patients could not be collected for comparison owing to large numbers (>7,000 referrals) and resource constraints.

Follow-up was performed using our unit's systems only, so subsequent cancers diagnosed in other units would not be identified. However, the next breast unit is over 90km away, making this unlikely to be significant.

Median follow-up was 17 months. Three years (mimicking the screening interval) would have been ideal, particularly as breast cancer can be slow growing, the age group attending the BFC are younger, and it could be many years before they are eligible for breast screening. However, this would delay our evaluation of this pathway for an excessive amount of time and we will continue to monitor the interval symptomatic cancer rate from this pathway as part of our ongoing pathway evaluation.

## Conclusions

We present a novel BFC pathway for managing patients referred to secondary care with EBS. Patients seen via this pathway had a low cancer detection rate, and there was a 0.06% symptomatic interval cancer rate. Implementation of the BFC pathway immediately improved the percentage of USC referrals seen within the 2WW standard. The BFC is a safe, well-utilised pathway that focuses limited resources on patients referred to secondary care who are most likely to have breast cancer.

## Funding

TH is funded by the National Institute of Health Research as an Academic Clinical Lecturer.

## Data availability statement

Data available on request from authors.
